# Coexistence patterns of soil methanogens are closely tied to methane generation and community assembly in rice paddies

**DOI:** 10.1186/s40168-020-00978-8

**Published:** 2021-01-22

**Authors:** Dong Li, Haowei Ni, Shuo Jiao, Yahai Lu, Jizhong Zhou, Bo Sun, Yuting Liang

**Affiliations:** 1 State Key Laboratory of Soil and Sustainable Agriculture, Institute of Soil Science, Chinese Academy of Sciences, Nanjing, 210008 China; 2 School of Environmental and Safety Engineering, Changzhou University, Changzhou, 213164 China; 3 University of the Chinese Academy of Sciences, Beijing, 100049 China; 4 State Key Laboratory of Crop Stress Biology in Arid Areas, College of Life Sciences, Northwest A&F University, Yangling, 712100 China; 5 College of Urban and Environmental Sciences, Peking University, Beijing, 100871 China; 6 Institute for Environmental Genomics, Department of Microbiology and Plant Biology, University of Oklahoma, Norman, OK 73019 USA

**Keywords:** CH_4_ emission, Methanogens, Co-occurrence network, Common and endemic coexistence, Stochastic and deterministic processes

## Abstract

**Background:**

Soil methanogens participate in complex interactions, which determine the community structures and functions. Studies continue to seek the coexistence patterns of soil methanogens, influencing factors and the contribution to methane (CH_4_) production, which are regulated primarily by species interactions, and the functional significance of these interactions. Here, methane emissions were measured in rice paddies across the Asian continent, and the complex interactions involved in coexistence patterns of methanogenic archaeal communities were represented as pairwise links in co-occurrence networks.

**Results:**

The network topological properties, which were positively correlated with mean annual temperature, were the most important predictor of CH_4_ emissions among all the biotic and abiotic factors. The methanogenic groups involved in commonly co-occurring links among the 39 local networks contributed most to CH_4_ emission (53.3%), much higher than the contribution of methanogenic groups with endemic links (36.8%). The potential keystone taxa, belonging to *Methanobacterium, Methanocella*, *Methanothrix*, and *Methanosarcina*, possessed high linkages with the methane generation functional genes *mcrA*, *fwdB*, *mtbA,* and *mtbC*. Moreover, the commonly coexisting taxa showed a very different assembly pattern, with ~ 30% determinism and ~ 70% stochasticity. In contrast, a higher proportion of stochasticity (93~99%) characterized the assembly of endemically coexisting taxa.

**Conclusions:**

These results suggest that the coexistence patterns of microbes are closely tied to their functional significance, and the potential importance of common coexistence further imply that complex networks of interactions may contribute more than species diversity to soil functions.

Video abstract

**Supplementary Information:**

The online version contains supplementary material available at 10.1186/s40168-020-00978-8.

## Introduction

Methanogens are a group of phylogenetically cohesive microbes from the domain Archaea that are responsible for the production of methane (CH_4_), which is regarded as the second most important greenhouse gas, with a global warming potential 25 times higher than that of CO_2_ [1]. The composition, distribution and functions of methanogenic and biogeographic communities have been widely studied from local to global scales [2–4]. The coexistence pattern of methanogenic communities is affected by environmental filters. For example, pH plays an important role in habitat filtering, which shapes the methanogenic biogeographic pattern in paddy soils, lakes, and dry lands [5, 6]. Temperature also affects the diversity and abundance of soil methanogenic archaea [7] as well as the carbon cycling and electron flow of complex methanogenic systems [8, 9]. CH_4_ emissions markedly increase with rising temperature and are linked to the transcriptional activities of methanogens [10]. Methanogens show significant metabolic flexibility during temperature adaptation [11, 12]. The community composition of methanogens that dominate the metabolic processes varies with temperature, resulting in changes in CH_4_ production [13].

The coexistence of methanogens is also a result of biotic interactions [14, 15]. Methanogens are engaged in complex associations including both inter- and intra-species syntrophic relationships and competition. Methanogenic archaea cooperate with syntrophic partners to obtain formate/H_2_ for CH_4_ synthesis. These partnerships are aggregated not only by metabolic interactions but also by additional amino acid auxotrophies [16]. However, detailed knowledge about complex species interactions in the field based on empirical studies is difficult to obtain for the most abundant and diverse microorganisms [17]. In recent decades, co-occurrence networks have become increasingly applied in ecology to infer microbial potential interactions [18, 19]. Co-abundance networks of root-associated methanogens were built to identify consortia associations that were involved in CH_4_ cycling in a field experiment [20]. Although coexistence cannot be strictly conflated with co-occurrence, co-occurrence relationships provide some support for elucidating potential coexistence patterns ranging from pairs of taxa to complex, multi-taxon communities in a variety of ecosystems [21–23].

In addition to these deterministic processes (environmental filtration and species interactions), it is broadly recognized that community assembly is simultaneously influenced by stochastic processes [24–26], including ecological drift, mutation, and random births and deaths [27]. Stochastic processes reveal a stronger effect in driving archaeal β-diversity in rice fields than in dryland [28]. Frequent flooding management may enhance ecological drift and dispersal limitation [28]. With the recognition of the critical importance of microbial coexistence, deciphering the coexistence patterns of soil methanogens and the underlying community assembly mechanism may help identify the potential keystones (microbial consortia) [20] responsible for CH_4_ production at large spatial scale.

Rice paddies are major man-made wetlands and rice agriculture is the largest anthropogenic source of CH_4_ emissions, with a range of 25-300 Tg CH_4_ per year [1]. To further understand the coexistence patterns and community assembly of methanogens and their linkage to CH_4_ production in typical paddy soils, 429 soil samples were collected from 39 rice paddies in 13 regions from northern to southern China to test the following hypotheses: (1) Complex co-occurrence relationships of methanogens are mediated by mean annual temperature (MAT) across continental rice paddies, and this coexistence pattern can partially predict variations in CH_4_ emissions. (2) A broadly distributed microbial communities that co-occur in different locations may play crucial roles in maintaining community and soil ecosystem functions [29, 30]. (3) Stochasticity dominates methanogenic community assembly but the importance of stochasticity and determinism differs between commonly and endemically coexisting taxa.

## Materials and methods

### Sampling and site characteristics

**Fig. 1:**
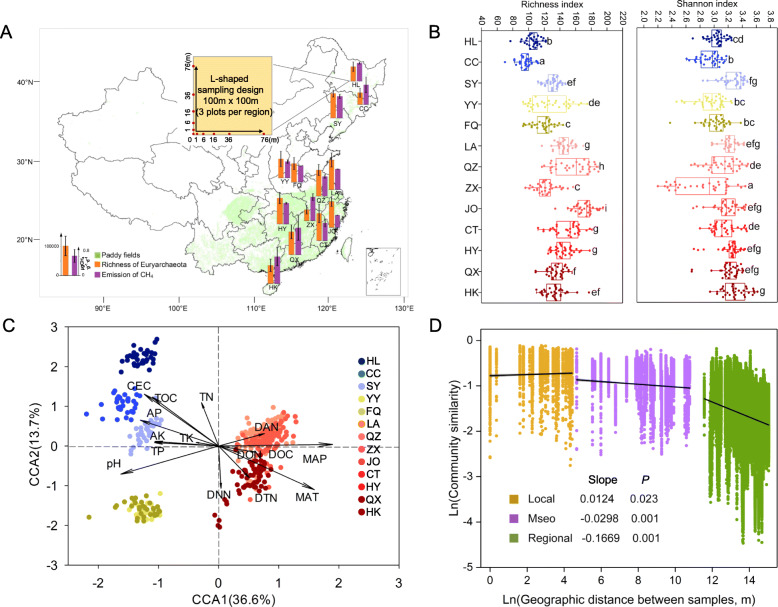
Methane generation potential and the distribution of methanogenic archaeal communities in paddy soils. **a** Richness (orange) of methanogenic archaeal communities and CH_4_ emission potential (purple) from 39 typical paddy fields (green) across northern to southern China. **b** α diversity (richness and Shannon index) of methanogens. The horizontal bars within boxes represent medians. The tops and bottoms of boxes represent 75th and 25th percentiles, respectively. **c** Canonical correspondence analysis (CCA) of methanogenic archaeal community structure. Black arrow indicates the vector of the explanatory variable, and points of different colors indicate paddy soil samples in 39 paddy fields (429 soil samples in total). MAT, mean annual temperature; MAP, mean annual precipitation; CEC, cation exchange capacity; TOC, total organic carbon; DOC, dissolved organic carbon; TN, total nitrogen; DTN, dissolved total nitrogen; DON, dissolved organic nitrogen; DAN, ammonium nitrogen; DNN, nitrate nitrogen; TP, total phosphorus; AP, available phosphorus; TK, total potassium; AK, available potassium. **d** Distance–decay relationships of methanogenic communities at three scales, local (1–100 m), meso (0.1–50 km), and regional (100–3500 km)

A total of 429 soil samples were taken from 39 paddy fields in 13 regions, covering a wide spatial range of 110° 10′ to 126° 14′ E and 19° 32′ to 46° 58′ N (Fig. 1a) during August to November 2013 after rice harvesting. The paddy fields represented four types of crop rotations (single rice, rice-wheat rotation, double rice, triple rice), five soil types (neutral black soil derived from loamy loess, alkaline fluvo-aquic soil derived from alluvial sediments of the Yellow River, hydromorphic paddy soils derived from sediments of lakes, acidic red soil derived from quaternary red clay, submergenic paddy soil derived from neritic sediment) (the geographic information of 13 sampling regions were listed in Table S1 including longitude, latitude, MAT and mean annual precipitation (MAP)). In each sampling field, 11 soil samples were taken within 100 m × 100 m plots using a spatially explicit “L-shaped” sampling design. Five cores with a diameter of 5 cm were randomly selected from the topsoil (0–15 cm), and each sub sample point was 0.5 m in diameter and mixed together (500 g in total). Soils were collected and sealed into sterile sampling bags, transported to the lab on ice. Then, the soils were divided into two subsamples in laboratory. One subsample was kept at 4 °C to measure the soil geochemical properties, and the other was stored at − 80 °C for molecular analyses. Because all the soil samples were collected after the rice harvest, this study did not consider the effects of aboveground crops.

Soil pH was determined with a glass electrode in the soil with a water-to-soil ratio of 2.5:1. Soil total organic carbon (SOC) and dissolved organic carbon (DOC) were measured by potassium dichromate oxidation. Total nitrogen (TN), nitrate (NO_3_^−^-N), and ammonium (NH_4_^+^-N) contents were determined by the Kjeldahl method. Total phosphorus (TP) and available phosphorus (AP) were measured with the sodium carbonate and Olsen-P methods, respectively. Total potassium (TK) and available potassium (AK) were measured by flame photometry after extraction with sodium hydroxide and ammonium acetate, respectively. The climatic data including mean annual temperature (MAT) and mean annual precipitation (MAP) were obtained from the Worldclim database (www.worldclim.org). All soil geochemical data are available in the repository Figshare (10.6084/m9.figshare.11493081.v2).

### Measurement of CH_4_ production potential

CH_4_ production potential was determined in laboratory [31]. The flasks of soil, with air vacuumed out, were purged with N_2_ repeatedly to remove residual CH_4_ and O_2_ and then incubated for approximately 50 h in darkness at 25 °C. Soil samples were taken with a pressure-lock syringe at 1 h and 50 h later, after the flasks were heavily shaken by hand, and analyzed for CH_4_ on GC-FID. CH_4_ production potential was calculated using the linear regression of the CH_4_ increase with incubation time and expressed in μg CH_4_ g^−1^ d^−1^. CH_4_ production potential was the average of the triplicates weighted by an interval of two adjacent measurements.

### Soil DNA extraction and MiSeq sequencing

Soil DNA was extracted from 2 g of well-mixed soil for each sample by combining freeze-grinding and sodium dodecyl sulfate for cell lysis as previously described [32]. The extracted DNA quality was assessed according to the 260/280 nm and 260/230 nm absorbance ratios with a NanoDrop 2000 instrument (Thermo Fisher Scientific, Wilmington, DE, USA). All DNA was stored at − 80 °C.

The methanogenic archaeal 16S rRNA gene was combined with adaptor sequences and barcode sequences by PCR amplification with the primer pair 1106F (TTWAGTCAGGCAACGAGC)/1378R (TGTGCAAGGAGCAGGGAC) [33]. Primer bias may be caused by chimeras, multi-template amplification bias or primer mismatch [34–36]. Previous studies evaluated this primer pair and showed that it is applicable to analysis of methanogenic archaeal community in paddy field soils by comparing different primer sets [37]. In addition, Feng et al. also used this primer pair to study the community composition of methanogens from paddy fields in China and showed that the primer could be used to study methanogenic Archaea in paddy soil of China [38]. An ABI GeneAmp® 9700 (ABI, Foster City, CA, USA) with a 20 μL reaction system containing 4 μL of 5× FastPfu Buffer, 0.8 μL of each primer (5 μM), 2 μL of 2.5 mM dNTPs, 10 ng of template DNA, and 0.4 μL of FastPfu polymerase was used to perform the PCR amplification. The qPCR program used for methanogenic archaea was 94 °C for 2 min, followed by 30 cycles of 94 °C for 30 s, 55 °C for 30 s, and 72 °C for 60 s, and subsequent extension and signal reading. The specificity of the amplification products was confirmed by melting curve analysis, and the expected sizes of the amplified fragments were checked in a 1.5% agarose gel. PCRs were conducted in triplicate for each sample. The results were combined after the PCR amplification. The PCR products were separated on a 2.0% agarose gel. We excised and purified the band of the correct size using an AxyPrep DNA Gel Extraction Kit (Axygen Scientific, Union City, CA, USA) and quantified with QuantiFluor™-ST (Promega, Madison, WI, USA).

The pooled DNA was diluted to 2 nM, loaded onto the reagent cartridge, and run on a MiSeq benchtop sequencer (Illumina Inc., San Diego, CA, USA). The samples were prepared for sequencing using a TruSeq DNA kit according to the manufacturer’s instructions. The purified mixture was diluted, denatured, re-diluted, mixed with PhiX (equal to 30% of the final DNA amount), and then submitted to an Illumina MiSeq system for sequencing with a Reagent Kit v2 2 × 250 bp, as described in the manufacturer’s manual.

Paired-end reads were first merged using FLASH and then quality filtered according to the procedure described by Caporaso et al. [39]. Chimera detection and removal were accomplished using the USEARCH tool in the UCHIME algorithm [40]. Sequences were split into groups according to taxonomy and assigned to OTUs at a 97% similarity level using the UPARSE pipeline [40]. Those OTUs lacking more than two sequences were removed; representative sequences of the remaining OTUs were assigned to taxonomic lineages using the RDP classifier within the SILVA database.

### GeoChip 5.0 experiments and raw data analyses

Generally, 600 ng of purified soil DNA from each sample was labeled with the fluorescent dye Cy-3 (GE Healthcare, CA, USA) using a random priming method as described previously [41], purified using a QIAquick Purification kit (Qiagen, CA, USA), and then dried in a SpeedVac (Thermo Savant, NY, USA) into a powder. Subsequently, the labeled DNA was resuspended in DNase/RNase-free distilled water and mixed completely with hybridization solution containing 1× Acgh blocking, 1× HI-RPM hybridization buffer, 10 pM universal standard DNA, 0.05 μg/μL Cot-1 DNA, and 10% formamide (final concentrations). Then, the solution was denatured at 95 °C for 3 min, incubated at 37 °C for 30 min, and hybridized with GeoChip 5.0 arrays (180 K) [42].

The microarray data were preprocessed by the microarray analysis pipeline on the IEG website (http://ieg.ou.edu/microarray/) as previously described [42]. The main steps were as follows: (i) remove the spots of poor quality, which were determined by a signal-to-noise ratio less than 2.0; (ii) calculate the relative abundance of each soil sample by dividing the total intensity of the detected probes, then multiply by a constant and take the natural logarithm transformation; and (iii) remove the detected probes in only two out of eight samples at the same sampling site.

### Data analyses

The *α*-diversity (richness and Shannon index) of each sample was calculated, and the *β*-diversity was estimated (based on Bray-Curtis distances between samples). The geographical distances among the sampling sites were calculated from the sampling coordinates. Canonical correspondence analysis (CCA) was performed to explore the relationships between the methanogenic community and major climate and edaphic variables. A forward selection procedure was used to select significant variables using the “ordiR2step” function from “vegan”. To estimate changes in *β*-diversity with distance at various scales, the slopes of distance decay relationship (DDR) [43] at three spatial scales were calculated: local scale (1–100 m), mesoscale (0.1–50 km), and regional scale (100–3500 km). The turnover rates were calculated as the slope of the linear least squares regression of the relationship between ln(community similarity) versus ln(geographic distance), and microbial similarity was calculated based on the 1 − Bray-Curtis distance. Spearman’s rank correlations were used to determine the relationship between environmental variables and network topological attributes. All analyses were conducted in R 3.6.1.

The co-occurrence networks of methanogens across all sites and in each field were constructed using CoNet [44], a robust ensemble-based network inference tool to detect nonrandom patterns of microbial co-occurrence using multiple correlation and similarity measures. Four methods were selected to evaluate pairwise associations among the OTUs: the Pearson, Spearman, Bray-Curtis, and Kullback-Leibler correlation methods. The initial thresholds for all four measures were selected to retrieve 1000 positive and 1000 negative edges. For each measure and edge, 1000 renormalized permutation and 1000 bootstrap scores were generated to alleviate compositionality bias. Connection retention was saved while satisfying the four methods. A measure-specific *P* value was computed first and then merged with Brown’s method. Edges with merged *P* values less than 0.05 were kept after multiple tests using the Benjamini-Hochberg procedure. The co-occurrence networks were visualized with Cytoscape 3.7.1. The network topological parameters were analyzed using Network Analyzer [45].

To include nonlinear relationships and multivariate interactions, a random forest classification analysis was performed to identify important predictors of CH_4_ emissions among multiple variables, including climatic and edaphic factors, microbial co-occurrence network and diversity. Random forest is a new classification and regression method that uses standard samples of training data and random feature selection in tree selection to modify standard classification and regression tree methods [46]. Random forest analysis evaluates the importance of each predictor by determine how much the mean square error (MSE) increases. The variables were selected when the predictor variables were randomly replaced and the other variables remain unchanged. Thus, MAT, MAP, TOC, TN, TP, microbial co-occurrence network, and diversity were included in the final random forest model. These analyses were performed using the “RandomForest” package in R, and the significance of both the model and each predictor was also assessed with the “rfUtilities” and “rfPermute” packages, respectively.

According to the frequency of the connection between the pairwise OTUs in 39 networks, we divided these pairwise OTUs into five groups: always endemic links, conditionally endemic links, moderate links, conditionally common links, and always common links (Table 1). The pairwise OTUs which categorized into conditionally common links (C. common) and always common links (A. common) were regarded as the keystones OTUs based on the random forest analysis (53.3% contribution to methane emission). Then, the network construction was conducted to analysis the relationship between keystone OTUs and methanogenic functional genes (via GeoChip 5.0) to understand the potential function of these keystones on methane emissions. For the annotation, the 16 s rRNA sequence from each related OTU were BLAST compared and searched at NCBI web site. To evaluate the phylogenetic community composition within each group, the mean nearest taxon distance (MNTD) for each sample was calculated as described previously [47]. To identify the processes driving soil microbial community composition within a sample, the standardized effect size measured MNTD (ses. MNTD), which quantifies the number of standard deviations of the observed MNTD values, was used to test for niche or dispersal limitations (999 randomizations). When the ses. MNTD values are negative and quantiles are low (*P* < 0.05), co-occurring species are more affected by phylogenetic clustering than by dispersal limitation. In this study, the ses. MNTD is the negative of the nearest taxon index (NTI) [48]. By contrast, positive values and high quantiles (*P* > 0.95) indicate that co-occurring species are more affected by dispersal limitation than by phylogenetic clustering. *β*MNTD is the abundance-weighted-mean phylogenetic distance among closest relatives occurring in two different communities, and *β*NTI is the number of standard deviations that the observed *β*MNTD is from the mean of the null distribution. *β*NTI values > 2 or ≤ − 2 indicate determinism in community assembly; in contrast, *β*NTI values between − 2 and 2 indicate stochasticity. All phylogenetic analyses were conducted using Picante in R [49].

**Table 1:** Five groups were classified based on the frequency of co-occurrence relationships between pairwise OTUs

	Edge numbers	Relative proportion	Related OTUs
Always endemic links (only appeared in 1 plot)	4049	41.82%	314
Conditionally endemic links (1 < plots number ≤ 3)	2928	30.24%	209
Moderate links (3 < plots ≤ 10)	2206	22.78%	131
Conditionally common links (10 < plots ≤ 20)	342	3.53%	32
Always common links (plots > 20)	157	1.62%	9

## Results

### Diversity of methanogenic communities in paddy soils

CH_4_ emissions varied considerably across rice paddies (Fig. 1a). The diversity dilution curves of all 429 samples indicated that the sequencing depth captured most of the microbial information (Fig. S1). The methanogenic α-diversity varied across sites (Fig. 1b) and was positively correlated with MAT (*P* < 0.05, ANOVA) (Fig. S2). CH_4_ emissions were correlated with Shannon-Wiener diversity of soil methanogens (Fig. S2).

The methanogenic communities were clustered separately by geographic locations along the different climatic zones (Fig. 1c). MAT, MAP, pH, CEC, and TOC were found to significantly influence methanogenic community distribution (*P* < 0.05). The definition of DDR means that the similarity of communities decreases with the increase of geographical distance. At the local scale, the slope of DDR is positive, which means the on a very small spatial scale (1–100 m), there is no DDR pattern for the methanogenic communities (Fig. 1d). Additionally, as the scale increased, the similarity of communities decreased, and the linear fitting slopes varied from − 0.0298 to − 0.1669. These results suggested that the methanogenic communities showed a clear biogeographic distribution pattern.

### Co-occurrence networks of methanogenic community

**Fig. 2:**
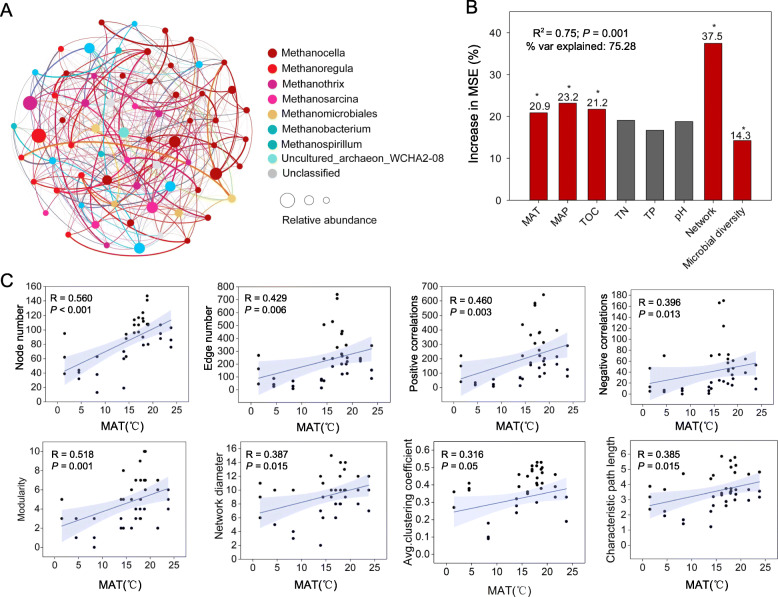
Co-occurrence networks and linkage with CH_4_ emissions. **a** The whole co-occurrence network structure of methanogenic community across all 39 paddy fields. The local co-occurrence network was also constructed in each field separately (Fig. S3). **b** The predictions of climatic variables (MAT, MAP), main soil geochemical variables (TOC, TN, TP, and pH), and methanogenic archaeal communities network and diversity to CH_4_ emissions base on random forest regression analysis. *R*^2^ means decision coefficient, and “% var explained” means the goodness of fit of the model. The red column indicates the factor that has a significant effect (*P* < 0.05; ANOVA, Duncan test), and the dark gray column indicates the factor that has no significant effect. The network index used in the model is the first component (56.9%) from the principal component analysis of eight main topological attributes of co-occurrence networks (node number, edge number, modularity, positive correlations, negative correlations, average clustering coefficient, network diameter, characteristic path length). Microbial diversity is Shannon index. **c** Spearman’s correlation analysis of network topological attributes and MAT. Blue shaded area is a 95% confidence interval. Correlations with other environmental factors are shown in Fig. S4

Networks of the methanogenic community were constructed at the OTU level across the all samples (Fig. 2a) and in each local plot (Fig. S3). The architecture of the local networks gradually became more complicated from north to south (Table S2, S4). The node and edge numbers increased from 39 to 147 and from 45 to 1039, respectively. *Methanoregula*, *Methanothrix*, *Methanocella*, *Methanosarcina*, *Methanobacterium* (genera), Methanomicrobiales, and Methanosarcinales (orders) accounted for a high proportion of the nodes, ranging from 62.2 (QZa) to 95.0% (HLb) (Fig. S3).

For the main environmental factors, MAT was the strongest predictor of the variations among eight network topological attributes (node number, edge number, positive correlations, negative correlations, modularity, average clustering coefficient, network diameter, characteristic path length) (Fig. S4). Linear regression analysis further indicated that MAT was positively correlated with all the network attributes (*P* < 0.05) (Fig. 2c, Fig. S4). In addition, we found that richness also has an impact on the network structure (Fig. S5). By controlling variables, the importance of temperature and richness to the network was explored, and it showed that temperature is more important (*R* = 0.254 > *R* = − 0.004) (Table S3). All findings suggested that MAT was more closely related than other environmental factors to the methanogenic networks structure.

### Commonly and endemically coexisting taxa of soil methanogens

Methanogenic community (co-occurrence network and diversity) factors associated with geochemical variables (MAT and MAP) and main soil factors (TOC, pH, TN and TP) explained 75.28% of total variations in CH_4_ emissions, of which methanogen interactions accounted for the greatest percentage (37.5%) (Fig. 2b). Linear regression analysis indicated that of the eight topological attributes, modularity, average clustering coefficient, and network diameter were positively correlated with CH_4_ emissions (*P* < 0.05) (Fig. S6). These results indicated that co-occurrence of these methanogens may be a major contributor to CH_4_ emissions.

**Fig. 3:**
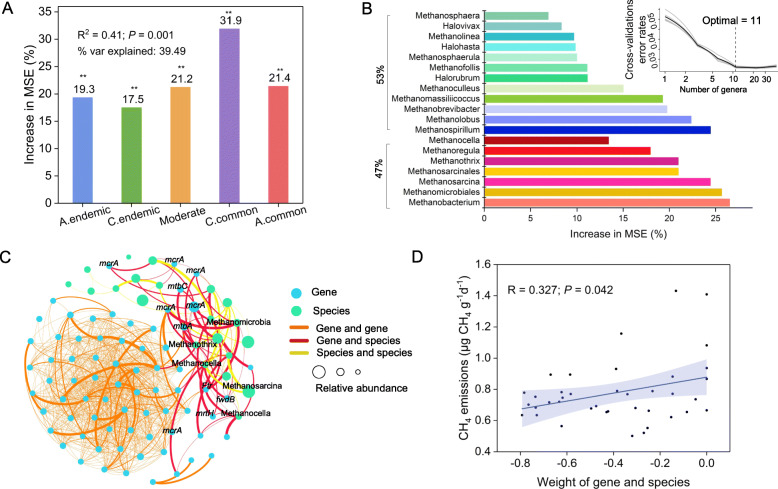
Potential keystone taxa for CH_4_ emissions. **a** Contribution of five groups of methanogenic communities to CH_4_ emissions based on random forest regression model. Five groups were classified based on the frequency of co-occurrence relationships between pairwise OTUs (A.endemic, always endemic group; C.endemic, conditionally endemic group; Moderate, moderate group; C.common, conditionally common group; A.common, always common group). **b** Biomarker taxa for CH_4_ emissions. The top 19 genera were identified by applying random forest classification of the relative abundance. Biomarker taxa are ranked in descending order of importance to the accuracy of the model. The inset represents tenfold cross-validation error as a function of the number of input genera used to differentiate microbiota in order of variable importance. Among the 19 genera, 7 genera contain the keystone OTUs (33 OTUs involved in C.common and A.common) and account for 47% of total microbial production of CH_4_ emissions; the other 12 genera account for 53%. **c** Functional co-occurrence network of 33 keystone OTUs and functional genes involved in CH_4_ generation. **d** Linear regression analysis of the connections between genes and species with CH_4_ emissions. Blue shaded area indicates 95% confidence interval (Spearman’s *P* < 0.05)

To address the hypothesis that broadly and locally co-occurring methanogens may play different roles in maintaining community structure and functions, the pairwise co-occurrence links of all 39 networks were classified into five groups based on the frequency with which the edge appeared among the 39 networks: always endemic, conditionally endemic, moderate, conditionally common, and always common links, with related OTU numbers of 314, 209, 131, 32, and 9, respectively (Table 1). The microbial richness and Shannon diversity of the five groups were significantly different (*P* < 0.05, ANOVA, Duncan test) (Fig. S7). A random forest model was constructed to predict the impacts of five groups of co-occurrence relationships on CH_4_ emissions (Fig. 3a). The contribution of common (conditionally and always common) co-occurrence relationships was the highest (53.3%), while that of endemic (conditionally and always endemic) co-occurrence relationships was 36.8%. Therefore, the 33 OTUs that commonly coexisted might be considered potential keystones for CH_4_ emissions (Table S5). Relative to their connection in the 429 soil samples, 17 OTUs were highly abundant species and 16 OTUs were rare species (Fig. S8). And we also found that the abundance distribution of these keystone species was related to temperature (Fig. S9), for example, *Methanomicrobiacea* at almost 20–25 °C has the highest abundance, *Methanosarcinaceae* and *Methanobacteriaceae* at 8–15 °C, *Methanocellaceae* at 8–19 °C is highest. To confirm this result, biomarker taxa for CH_4_ emissions were screened at the genus level (Fig. 3b). Among the top 19 biomarker genera with relatively high impact, those potential keystone taxa were included in 7 genera that accounted 47% of total microbial production of CH_4_ emissions.

The functional network was constructed among the 33 potential keystones and methane generation functional genes detected by GeoChip (Fig. 3c, Fig. S10). In total, there were 1119 edges among 92 nodes. Intensive relationships were observed among keystones and functional genes. The sum of the edge weights between gene and gene, species and gene, and species and species were 1.26, 1.01, and 0.40, respectively, indicating that there were relatively strong links between keystones and functional genes. The genes and species involved in these interactions were mainly *mcrA* (methyl-coenzyme M reductase alpha subunit), *fwdB* (molybdenum/tungsten formylmethanofuran dehydrogenases), *mtbA* (methylcobalamin: coenzyme M methyltransferase), *mtbC* (B12 binding domain of corrinoid proteins), *Methanocella*, *Methanothrix*, *Methanosarcina*, and *Methanobacterium*. Linear regression analysis showed that the weight of species and genes linkages was positively correlated with CH_4_ emissions (*P* < 0.05) (Fig. 3d).

### Different community assembly of commonly and endemically coexisting taxa

**Fig. 4:**
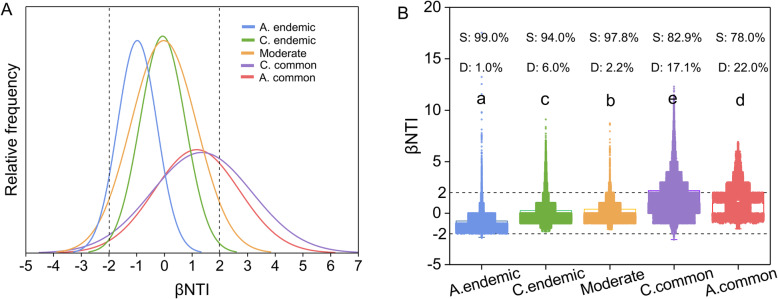
Community assembly of five groups of methanogenic communities. **a** The distribution of beta nearest taxon index (*β*NTI). Each observation is the number of null model standard deviations. The observed value is from the mean of its associated null distribution. **b** Box-plot of the total *β*NTI of five groups. S, stochastic process; D, deterministic process. Boxes followed by different letters differed significantly at *P* < 0.05 (ANOVA, Duncan test)

The beta nearest taxon index (*β*NTI) values of the five groups were mainly between − 2 and 2 (Table 2): always endemic (98.76%), conditionally endemic (93.2%), moderate (97.58%), conditionally common (74.21%), and always common (71.52%). The community assembly was dominated by stochastic processes, while the proportion of deterministic processes increased for common coexistence taxa. The distributions of *β*NTI values of common coexistence taxa were more extensive and had a relatively low frequency (Fig. 4). The *β*NTI of other groups centralized between 2 and − 2 with a relatively higher frequency.

**Table 2:** The relative contribution (%) of deterministic and stochastic processes to community assembly of five groups with OTUs related to endemic, moderate, and common links

	Determinism (%)	Stochasticity (%)
Always endemic group	1.24	98.76
Conditionally endemic group	6.80	93.20
Moderate group	2.42	97.58
Conditionally common group	28.48	71.52
Always common group	25.79	74.21

## Discussion

Using network analysis to explore the direct and/or indirect cooperation between microbial taxa coexisting across complex and diverse communities could help to ascertain the functional roles and assembly processes in the basic ecology and life history strategies of many microbiota [18, 50, 51]. In the present study, in accordance with our hypothesis that intricate co-occurrence relationships of methanogens are mediated by MAT across continental rice paddies, we found that all the topological attributes of the network were positively correlated with MAT. The increasing average clustering coefficient and characteristic path length indicated that highly connected OTUs were grouped in their neighborhood and clustered together rather than randomly [18]. One explanation is that the temperature shapes distinct community composition of methanogens in long-term rice paddies [28]. Based on the metabolic theory of ecology [52] and latitudinal diversity gradient, temperature increase soil microbial richness [53], and spatial heterogeneity [54], the structure of both archaeal and bacterial communities involved in the turnover of acetate and propionate in methanogenic rice field soil varied with the gradient temperature from 25 to 50 °C [55]. Another potential explanation for the topological change is that environmental filtering affects microbial competition and mutualism [18]. Adaptation to environmental stress in one species of microbe may increase/decrease selection pressure on another species, giving rise to antagonistic/sympathetic cooperative interaction [51]. Our results indicated that MAT had a higher correlation coefficient with positive interactions of methanogens rather than negative interactions. Temperature may enhance the cooperative interactions of methanogenic archaea, which have an important effect on community functions in rice paddies. In the present study, the coexistence relationship can better predict the variations of CH_4_ emissions compared with microbial diversity (Fig. 2b). The result was consistent with the previously network-based research that the uptake of carbon by the soil food web increases from 50 to 75% when the network connectance index increased from 0.626 to 1.278, suggesting that network structure is tightly related to the ecosystem functional process [56]. The tightening connection between taxa might help to reveal the potential niche occupancy characteristics shared by community members [18]. Furthermore, the long-term geological processes impart lasting legacies on the contemporary environments [57]. Geological processes directly or indirectly affect biodiversity and ecosystem functions. For example, previous study revealed that the regional-scale variation of climate change can determine the effects of biodiversity on ecosystem multifunctionality in natural ecosystems [58]. Hu et al. explored the key drivers of biological community and found that MAT had the strongest influence on bacterial communities [57]. In addition, geological processes may lead to the biological speciation and evolution [59, 60]. Poltak et al. proposed an evolutionary scenario, in which the common ancestor of Archaea harbored the ability for methane metabolism (including the evolution of methyl-coenzyme M reductase-containing hot spring Archaea) [61, 62]. Coevolution among species can enhance ecosystem characteristics; for example, species evolve complementary resource utilization, thus improving ecosystem productivity [61].

The roles of global trends (generalist edges) and local signals (specialist edges) in adaptations to environmental factors accompanied by coexistence patterns in antagonistic/sympathetic cooperative interactions are essential in research on co-occurrence patterns [51, 63]. In the present study, more than 72% of the edges were identified as endemic edges (the frequency of edge numbers ≤ 3 among the 39 networks), which included over 75% of OTUs in co-occurrence relationships in the methanogenic community (Table 1). However, the contribution of endemic links to methanogenesis (36.8%) was far lower than the contribution of common links (53.3%). This result conflicts with the observation that specialists consume resources more rapidly than generalists [64].

Based on the occupancy scenario (i.e., habitat generalists and habitat specialists) [18], we further seek to categorize the potential keystone taxa. The potential keystone species involved in common links belong to *Methanosarcinaceae*, *Methanocellales*, *Methanobacteriales,* and *Methanomicrobiales*. These taxa may have strong adaptability in maintaining important common relationships in the community. For example, *Methanosarcinaceae* can also use H_2_/CO_2_ as substrates, although not as effective as *Methanobacteriaceae* and *Methanocellaceae* [65]. *Methanosarcinaceae includes acetoclastic methanogens and other generalist methanogens, and Methanocellaceae, Methanomicrobiacea, Methanoregulaceae, and Methanobacteriaceae are all hydrogenotrophic* [1]. Acetate formed by acetogenic bacteria can be either used directly by some methanogens (*Methanosarcina* spp. and *Methanosaeta* spp.) and can also be degraded by syntrophic associations of bacteria (e.g., syntrophic acetate oxidizers) and hydrogen-consuming methanogenic archaea [66]. Hydrogenotrophic methanogenesis could act as the sinks for electron through interspecies electron transfer that reduce equivalents between hydrogen-forming acetogenic bacteria and hydrogen-consuming Archaea [67]. In a previous study, hydrogenotrophic methanogens (e.g., *Methanoregula* and *Methanocella*) can sustain in low dissolved H_2_ concentrations and produce acetate for *Methanosarcina* [68]. Temperature affects the overall diversity of the microbial communities. Previous research confirmed that different groups of methanogens could become predominant at different temperatures [69]. Most known methanogens were mesophilic and moderate to extreme thermophilic archaea [70]. For example, *Methanobacteriace*ae showed the greatest activity at 30 °C, and *Methanocellaceae* were favored in the late stages at 45 °C. For the acetoclastic methanogens, *Methanosarcinaceae* could produce CH_4_ via both hydrogenotrophic and acetoclastic processes under moderate temperatures (10–30 °C) and exclusively consumed H_2_/CO_2_ rather than acetate at higher temperatures (45 °C). Their abundance is highest in the range of 8–21.6 °C (Fig. S9). Compared with the optimum growth temperature reported in the literature, they are not exactly the same. We propose that such flexible strategies give the rise to the shift of ecosystem processes and functions, subsequently increasing the efficiency of CH_4_ production.

We also found that the keystone taxa possessed high linkages with functional genes *mcrA*, *fwdB*, *mtbA,* and *mtbC* involved in the methanogenesis process (Fig. 3c). Methylcobamide: CoM methyltransferase (*mtbA*) is involved in CO_2_ reduction to methane and acetate disproportionation into methane and CO_2_ in methylotrophic methanogenesis [65]*.* These processes may be the reasons that keystone species dominate methane emissions. The functional genes involved in this study are all related to methane production (21 functional genes), which have been extensively studied and verified in previous studies, such as *mcrA*, *fwdB*, and *mtbA*. In present study, GeoChip microarray was used to measure the abundance of functional genes include methane metabolism process. Therefore, a greater number of available genes could identify by used of network analysis because the GeoChip is a close loop system that cannot discover new functional genes (unless such new gene had been discovered and put into the GeoChip library). So, all the functional and keystone genes that we have screened out based on network analysis, and statistically and analytically confirmed. We hope to identify more genes related to the structure and function of methanogens in future studies.

The community assembly processes with the linkage to CH_4_ production are simultaneously influenced by deterministic and stochastic processes [71]. Co-occurring species adapt to environmental conditions by generating a trade-off between environmental filtering and disposal limitation and thereby alter the selection pressures on other species and how they use the available resources [72, 73]. Species sorting is the deterministic process which is defined as the ecological forces that alter the community structure due to the fitness differences among organisms and environmental heterogeneity. Conversely, dispersal limitation could either be deterministic, stochastic, or both [74]. Jiao et al. reported that co-occurrence associations of archaea tends to be more frequent in low-latitude rice paddies because of species sorting [75]. Since the MAT was the dominant environmental filter in the present study, we found that the distribution of *β*NTI shifted to the edges (*β*NTI = 2) with the increased frequency of co-occurrence relationships. The relative contribution of deterministic processes to community assembly had a similar tendency accompanied by an increase in co-occurrence relationships (Table 2). Although the stochastic processes still play a dominant role in driving microbial community assembly, our result demonstrated that the commonly coexisting taxa undergoing strong environmental selection (MAT) and adaptations for survival are more likely to be associated together and play a more important role in CH_4_ emissions. This inference could be validated by a previous study showing that the biotic selection (species sorting) contributed more to microbial assembly processes than other forces in paddy soils [76]. Since methanogenesis is subject to distinct temperature filtering, the closer coexistence relationship of commonly coexisting taxa would appear with stronger niche occupation [77], consequently improving the efficiency of CH_4_ production. In the future research, such coexistence relationship may be considered to add in climate warming model (e.g., GISS global climate models) to improve the accuracy of model prediction.

Furthermore, the effects of rice at different development stages on the community composition of methanogenic archaea will affect methane emission. Kimura et al. reported that the types and amounts of various compounds supplied by rice roots to rhizospheres varied with different growth stages [78]. In present study, some of the keystone taxa, such as Methanosarcinaceae, Methanobacteriales, and Methanomicrobiales, are consistent with the previous research that these keystone methanogens during the rice growth period that significantly affect methane emission in paddies [79–81]. Therefore, although we did not consider the effect of crops on the composition and function of methanogens in present study, the role of crops cannot be ignored. In the future research, we need to study the role of these keystone species on methane emissions at different stages of rice growth and to analyze the effects of rice growth on these microbial communities, structures, and functions.

## Conclusion

In conclusion, methanogenic co-occurrence patterns were studied across rice paddies at a continental scale, and tightened network structure was found to be highly mediated by MAT. Common co-occurrence relationships may be more important than endemic co-occurrence relationships to the function of microorganisms in CH_4_ production. The relative importance of stochastic processes and deterministic processes differed between community assemblies of taxa that commonly coexisted and those that endemically coexisted. These results suggest that the microbial coexistence patterns are closely tied to the functional significance of the community, with particular importance of commonly coexisting taxa, further indicating that complex networks of interaction may contribute more than species diversity to soil functions. Both field and laboratory experiments are required to further address the methanogens coexistence pattern that drives community composition and functions at ecological timescales as well as for the evolution of species interactions.

## Supplementary Information

**Additional file 1: Figure S1.** Sampling and sequencing of methanogens. Abscissa: the number of sequences randomly selected from the sample; ordinate: the species diversity and the number of OTU that can be represented by the random sampling sequence. Each curve in the figure represents a sample, marked with a different color. **Figure S2.** Linear regression of methanogenic archaea α-diversity and MAT (A) and CH_4_ emissions (B). Solid lines denote significant relationship with 95% confidence interval (shadow area). Dotted line denotes *P* > 0.05. **Figure S3.** The co-occurrence network structure of methanogenic community of 39 plots. Percentage in the top right corner indicates the proportion of 33 key OTUs (**Table S4**) in total node numbers of each network. **Figure S4.** Contributions of climatic variables and soil properties to the network topological attributes based on correlation and random forest regression model. Circle size represents the variable importance (that is, proportion of explained variability calculated via forest regression analysis analysis). Colors represent Spearman’s correlation coefficients (node: node number; edge: edge number; modularity: modularity; positive: positive correlations; negative: negative correlations; Avg.C.C: average clustering coefficient; N.D: network diameter; C.P.L: characteristic path length). **Figure S5.** Spearman’s correlation analysis of network topological attributes and richness. Blue shaded area is a 95% confidence interval. **Figure S6.** Spearman’s correlation analysis of network topological attributes and CH_4_ emissions. Dotted lines denote *P* > 0.05. Solid lines indicate a significant correlation (*P* < 0.05), with the blue shaded area showing the 95% confidence interval. **Figure S7.** Relative abundance of OTUs of the five groups of methanogenic archaeal communities. Each column followed by different letters differed significantly at *P* < 0.05 (ANOVA, Duncan test). (A.endemic: always endemic group; C.endemic: conditionally endemic group; C.common: conditionally common group; A.common: always common group). **Figure S8.** Relative abundance frequency histogram of 33 keystone OTUs in 429 samples. The blue histogram indicates rare OTUs (average relative abundance < 1% in 429 samples). The red histogram indicates abundant OTUs (average relative abundance > 1% in 429 samples), and the inset represents low-frequency OTUs with average relative abundance < 1%. **Figure S9.** The relative abundance of keystone taxa (at the family level) changes with temperature. (HL: 1.5°C, CC: 4.5°C, SY:8.3°C, YY: 14.4°C, FQ:13.9°C, LA: 16.1°C, QZ:17.9°C, ZX:17°C, JO:18.8°C, CT:19°C, HY:18°C, QX:21.6°C, HK:23.8°C). **Figure S10.** The relationship between keystone species and microbial functional genes related to methane generation depicted as colored segments in a CIRCOS plot. Ribbons connecting two segments indicate the interaction between the two. The size of the ribbon is proportional to the number of links. **Table S1.** Geographic information of 13 sampling regions with 3 plots in each region. **Table S2.** The topological features of the co-occurrence network in each plot. **Table S3.** Partial mantel test between network topology attributes and variables. **Table S4.** Changes of network topology attributes with latitude. **Table S5.** Information of 33 keystone OTUs involved in common co-occurrence relationship.

## Data Availability

The raw sequence data reported in this paper have been deposited in the Genome Sequence Archive in BIG Data Center, Chinese Academy of Sciences, under accession number CRA001914 that are publicly accessible at http://bigd.big.ac.cn/gsa. The GeoChip data is available in the repository Figshare, 10.6084/m9.figshare.9746303. The sampling information and soil geochemical data are also available in the repository Figshare, 10.6084/m9.figshare.11493081.v2.
