# Supplementary material for: Coexistence patterns of soil methanogens are closely tied to methane generation and community assembly in rice paddies
Source: Microbiome. 2021 Jan 22;9:20. doi: 10.1186/s40168-020-00978-8 (PMC7825242; doi:10.1186/s40168-020-00978-8)
Supplement: Supplementary file 2 — Additional file 1: Figure S1. Sampling and sequencing of methanogens. Abscissa: the number of sequences randomly selected from the sample; ordinate: the species diversity and the number of OTU that can be represented by the random sampling sequence. Each curve in the figure represents a sample, marked with a different color. Figure S2. Linear regression of methanogenic archaea α-diversity and MAT (A) and CH4 emissions (B). Solid lines denote significant relationship with 95% confidence interval (shadow area). Dotted line denotes P > 0.05. Figure S3. The co-occurrence network structure of methanogenic community of 39 plots. Percentage in the top right corner indicates the proportion of 33 key OTUs (Table S4) in total node numbers of each network. Figure S4. Contributions of climatic variables and soil properties to the network topological attributes based on correlation and random forest regression model. Circle size represents the variable importance (that is, proportion of explained variability calculated via forest regression analysis analysis). Colors represent Spearman’s correlation coefficients (node: node number; edge: edge number; modularity: modularity; positive: positive correlations; negative: negative correlations; Avg.C.C: average clustering coefficient; N.D: network diameter; C.P.L: characteristic path length). Figure S5. Spearman’s correlation analysis of network topological attributes and richness. Blue shaded area is a 95% confidence interval. Figure S6. Spearman’s correlation analysis of network topological attributes and CH4 emissions. Dotted lines denote P > 0.05. Solid lines indicate a significant correlation (P < 0.05), with the blue shaded area showing the 95% confidence interval. Figure S7. Relative abundance of OTUs of the five groups of methanogenic archaeal communities. Each column followed by different letters differed significantly at P < 0.05 (ANOVA, Duncan test). (A.endemic: always endemic group; C.endemic: conditionally endemic group; [file 40168_2020_978_MOESM2_ESM.docx]

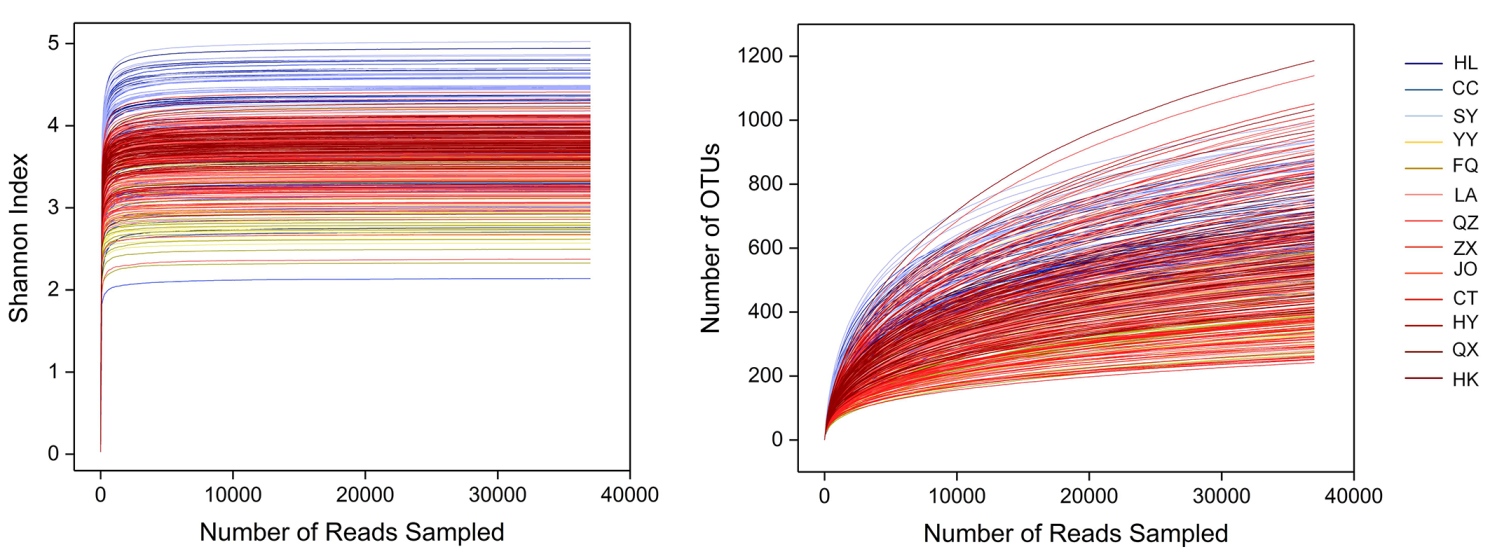


**Fig. S1** Sampling and sequencing of methanogens. Abscissa: the number of sequences randomly selected from the sample; ordinate: the species diversity and the number of OTU that can be represented by the random sampling sequence. Each curve in the figure represents a sample, marked with a different color.


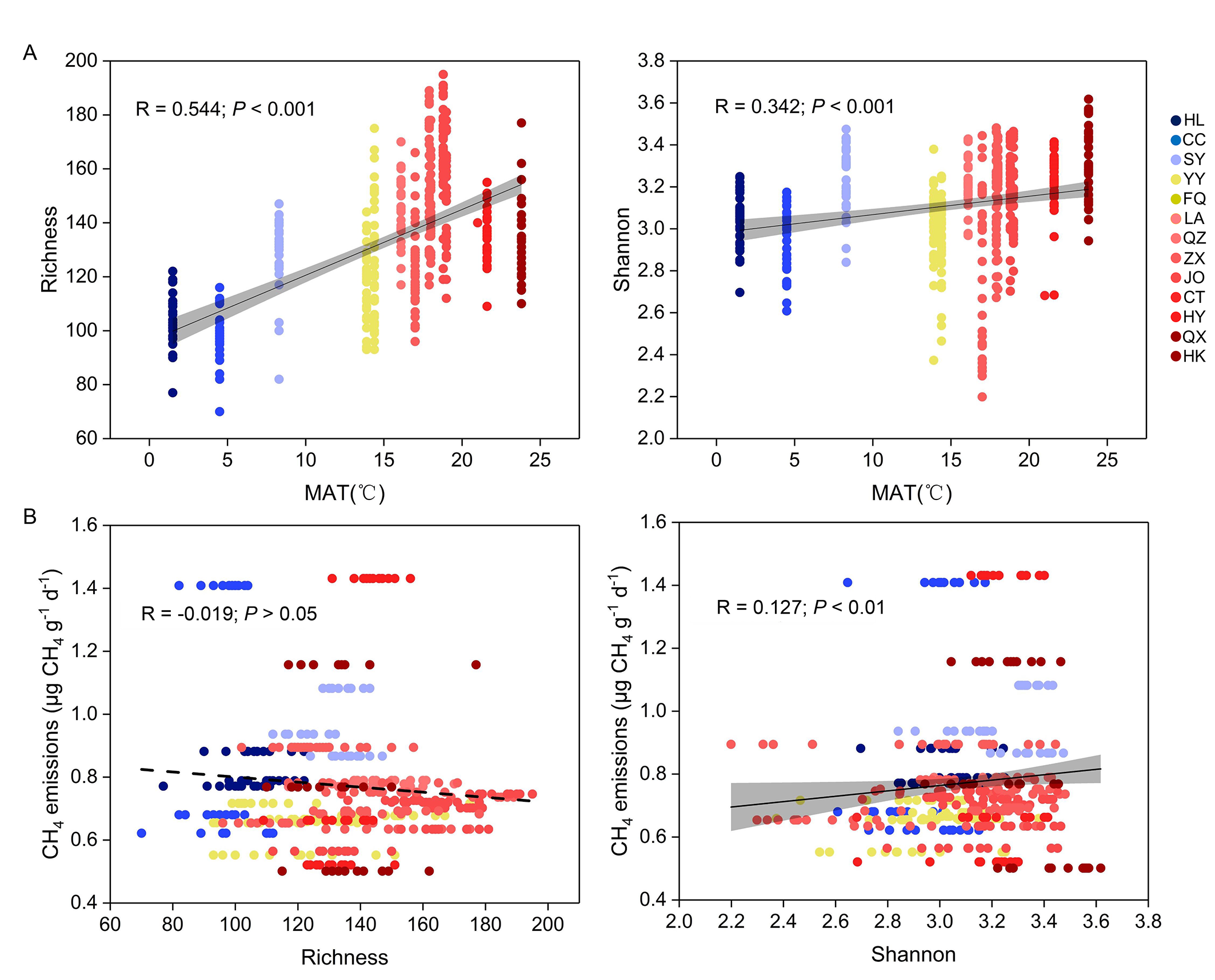


**Fig. S2** Linear regression of methanogenic archaea α-diversity and MAT (A) and CH_4_ emissions (B). Solid lines denote significant relationship with 95% confidence interval (shadow area). Dotted line denotes *P* > 0.05.


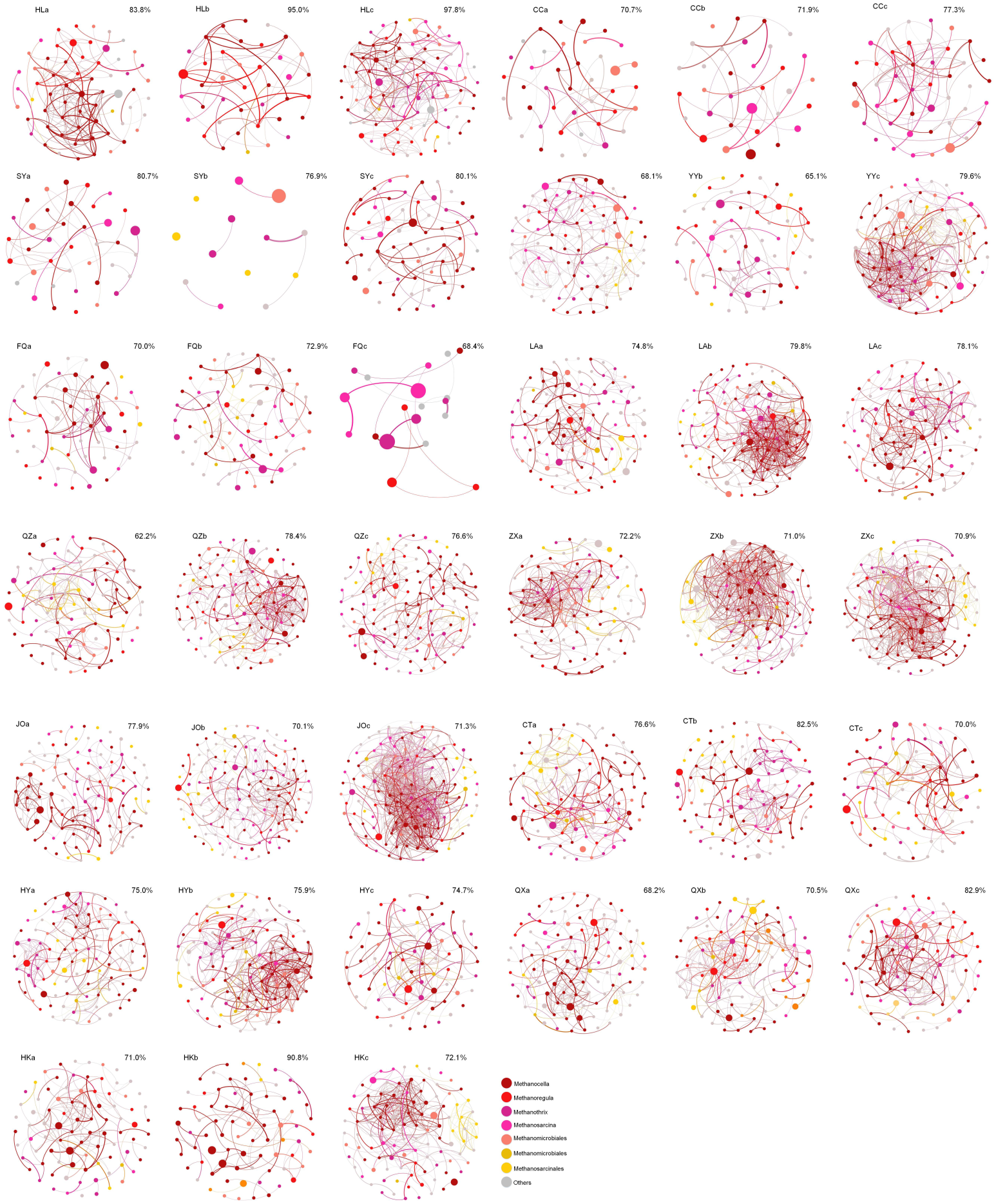
**Fig. S3** The co-occurrence network structure of methanogenic community of 39 plots. Percentage in the top right corner indicates the proportion of 33 key OTUs (Table S4) in total node numbers of each network.


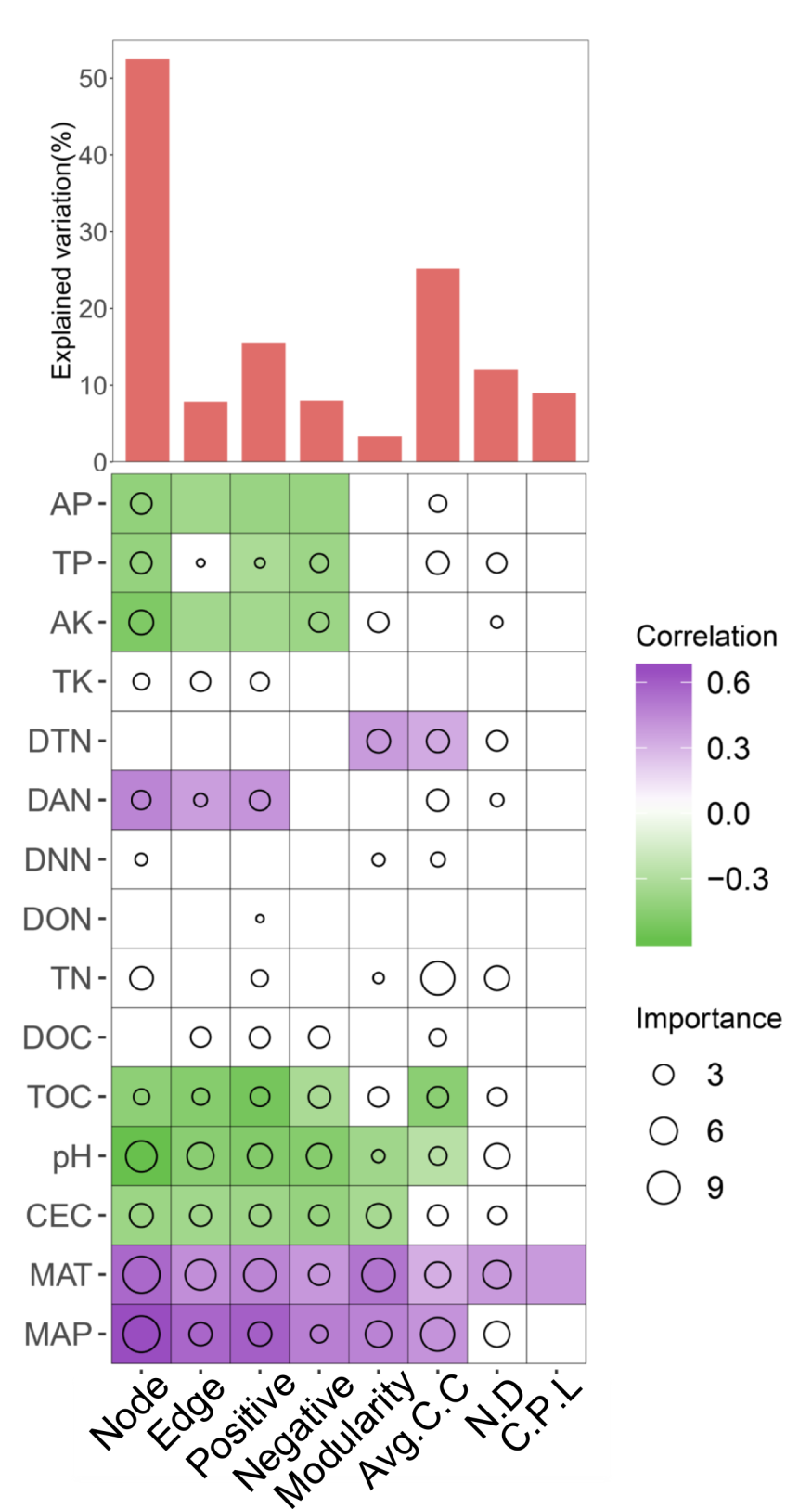


**Fig. S4** Contributions of climatic variables and soil properties to the network topological attributes based on correlation and random forest regression model. Circle size represents the variable importance (that is, proportion of explained variability calculated via forest regression analysis analysis). Colors represent Spearman’s correlation coefficients (node: node number; edge: edge number; modularity: modularity; positive: positive correlations; negative: negative correlations; Avg.C.C: average clustering coefficient; N.D: network diameter; C.P.L: characteristic path length).





**Fig. S5** Spearman’s correlation analysis of network topological attributes and richness. Blue shaded area is a 95% confidence interval.


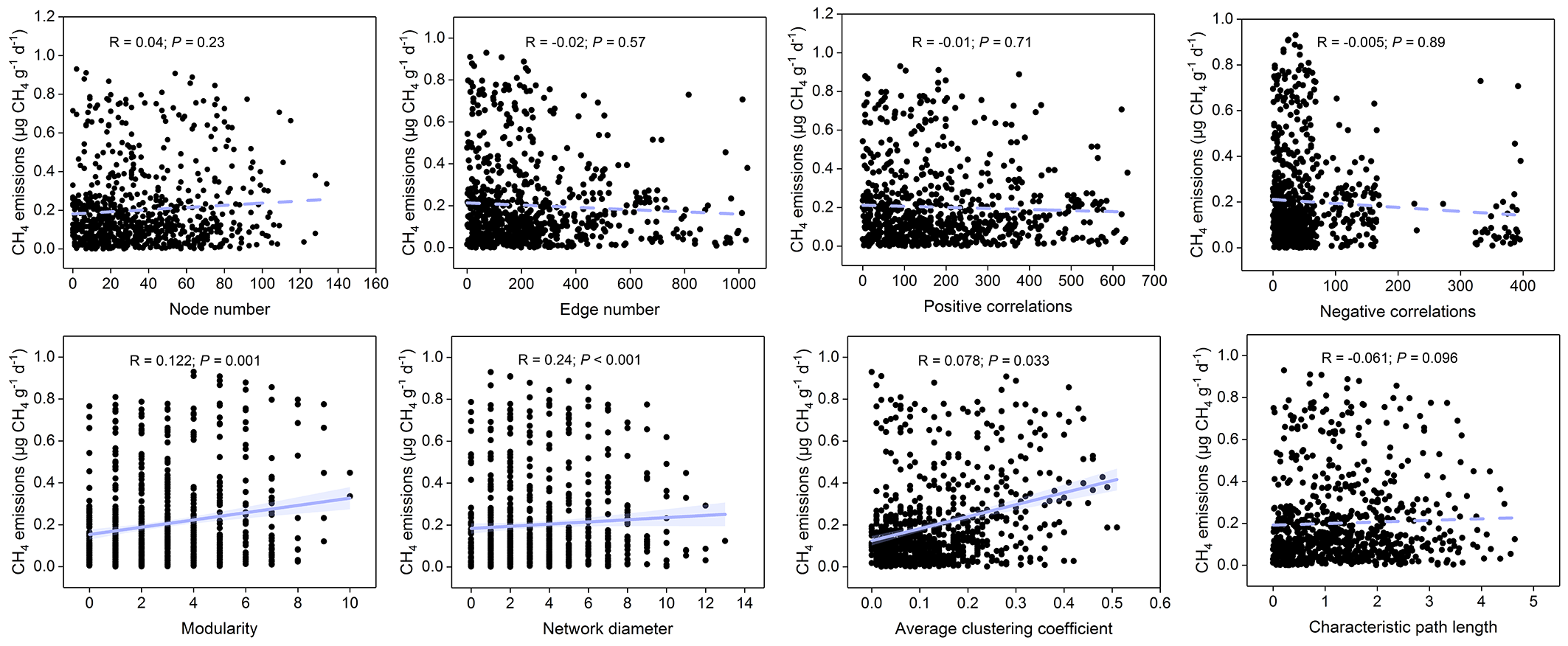


**Fig. S6** Spearman’s correlation analysis of network topological attributes and CH_4_ emissions. Dotted lines denote *P* > 0.05. Solid lines indicate a significant correlation (*P* < 0.05), with the blue shaded area showing the 95% confidence interval.

**
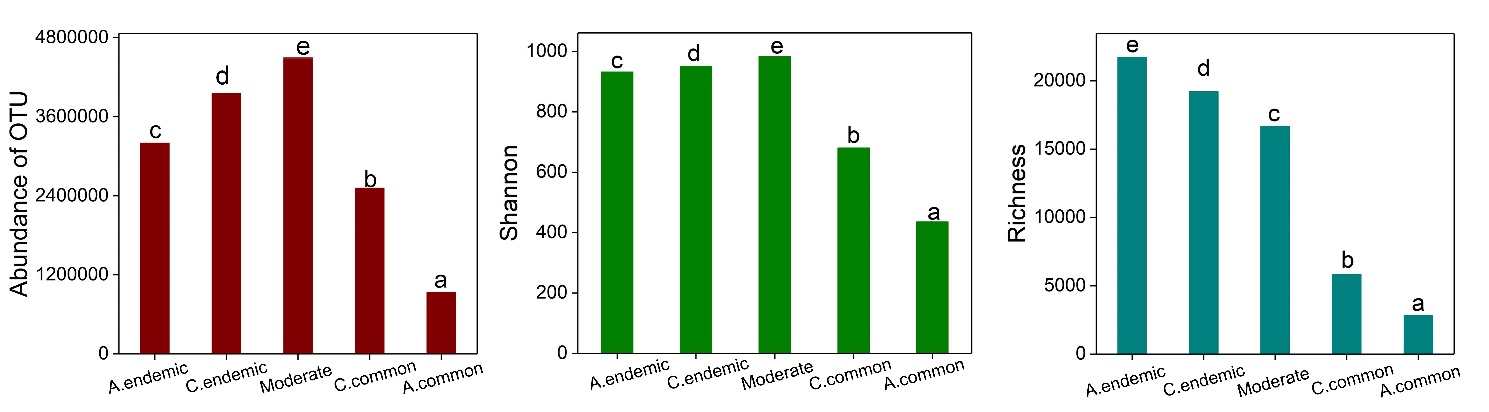
**

**Fig. S7** Relative abundance of OTUs of the five groups of methanogenic archaeal communities. Each column followed by different letters differed significantly at *P* < 0.05 (ANOVA, Duncan test). (A.endemic: always endemic group; C.endemic: conditionally endemic group; C.common: conditionally common group; A.common: always common group).


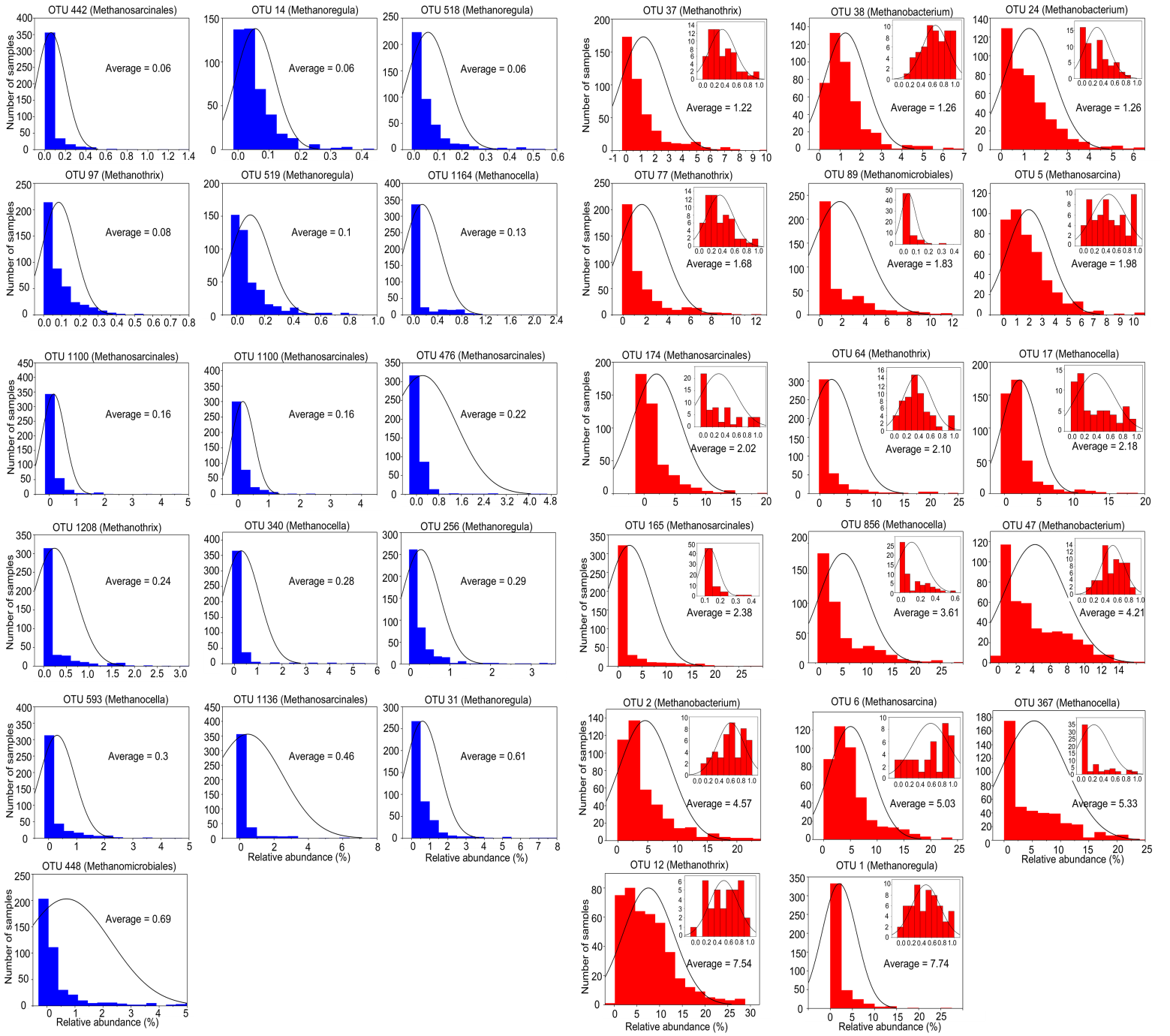
**Fig. S8** Relative abundance frequency histogram of 33 keystone OTUs in 429 samples. The blue histogram indicates rare OTUs (average relative abundance < 1% in 429 samples). The red histogram indicates abundant OTUs (average relative abundance > 1% in 429 samples), and the inset represents low-frequency OTUs with average relative abundance < 1%.


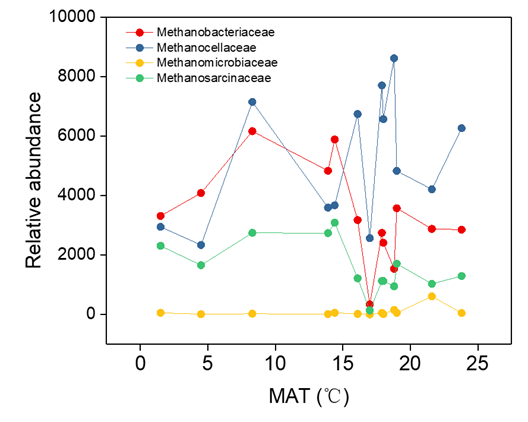


**Fig. S9** The relative abundance of keystone taxa (at the family level) changes with temperature. (HL: 1.5℃, CC: 4.5℃, SY:8.3℃, YY: 14.4℃, FQ:13.9℃, LA: 16.1℃, QZ:17.9℃, ZX:17℃, JO:18.8℃, CT:19℃, HY:18℃, QX:21.6℃, HK:23.8℃)


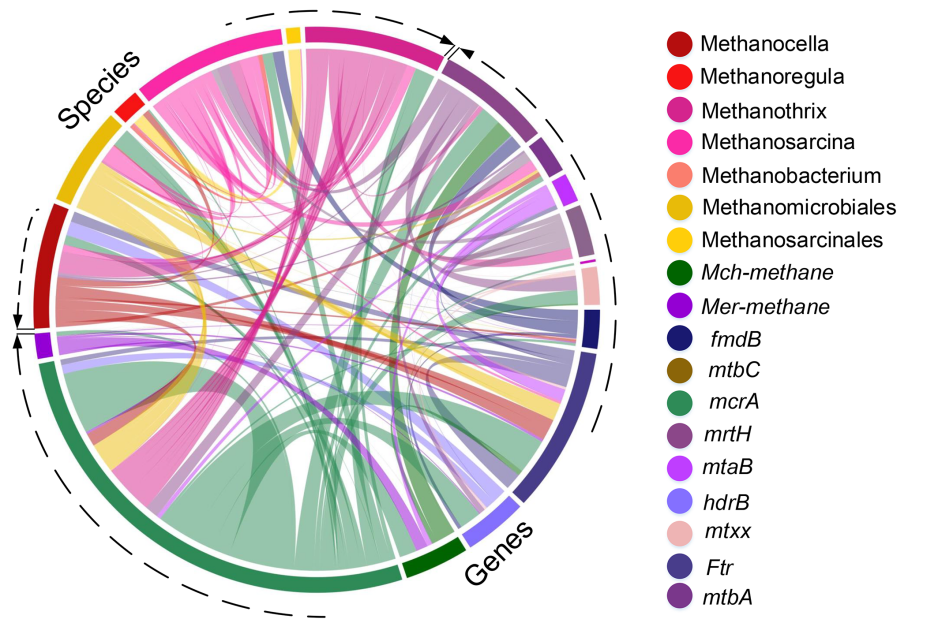


**Fig. S10** The relationship between keystone species and microbial functional genes related to methane generation depicted as colored segments in a CIRCOS plot. Ribbons connecting two segments indicate the interaction between the two. The size of the ribbon is proportional to the number of links.

**Table S1** Geographic information of 13 sampling regions with 3 plots in each region.

|  | Longitude | Latitude | MAT(^o^C) | MAP (mm) |
| --- | --- | --- | --- | --- |
| HL (Hailun) | 126.97°E | 47.82°N | 1.5 | 556 |
| CC (Changchun) | 125.35°E | 44.74°N | 4.5 | 520 |
| SY (Shenyang) | 123.86°E | 41.57°N | 8.3 | 400 |
| YY (Yuanyang) | 113.36°E | 35.14°N | 14.4 | 550 |
| FQ (Fengqiu) | 114.85°E | 34.89°N | 13.9 | 615 |
| LA (Linan) | 116.52°E | 30.61°N | 16.1 | 1613 |
| QZ (Quzhou) | 118.91°E | 27.24°N | 17.9 | 1971 |
| ZX (Zixi) | 117.92°E | 27.75°N | 17 | 1934 |
| JO (Jian’ou) | 118.72°E | 27.05°N | 18.8 | 1664 |
| CT (Changting) | 116.52°E | 26.61°N | 19 | 1700 |
| HY (Hengyang) | 112.18°E | 25.52°N | 18 | 1500 |
| QX (Qingxin) | 112.72°E | 23.53°N | 21.6 | 2215 |
| HK (Haikou) | 110.01°E | 19.72°N | 23.8 | 1664 |

MAT, mean annual temperature; MAP, mean annual precipitation; All the soil geochemical data are available in the repository Figshare https://doi.org/10.6084/m9.figshare.11493081.v2.

**Table S2** The topological features of the co-occurrence network in each plot.

| Sites | Node | Edge | Positive | Negative | Modularity | Average clustering  coefficient | Network  diameter | Characteristic  path length |
| --- | --- | --- | --- | --- | --- | --- | --- | --- |
| HLa | 62 | 163 | 150 | 13 | 3 | 0.36 | 6 | 2.38 |
| HLb | 39 | 45 | 40 | 5 | 3 | 0.27 | 9 | 3.19 |
| HLc | 95 | 268 | 221 | 47 | 5 | 0.36 | 11 | 3.88 |
| CCa | 41 | 41 | 34 | 7 | 3 | 0.37 | 5 | 1.94 |
| CCb | 32 | 26 | 22 | 4 | 1 | 0.38 | 5 | 2.24 |
| CCc | 44 | 88 | 18 | 70 | 3 | 0.41 | 10 | 3.68 |
| SYa | 38 | 28 | 22 | 6 | 1 | 0.09 | 4 | 1.68 |
| SYb | 13 | 8 | 8 | 0 | 0 | 0.10 | 3 | 1.42 |
| SYc | 63 | 68 | 58 | 10 | 3 | 0.18 | 10 | 4.71 |
| YYa | 94 | 242 | 221 | 21 | 8 | 0.46 | 11 | 4.61 |
| YYb | 63 | 70 | 60 | 10 | 5 | 0.39 | 9 | 3.20 |
| YYc | 88 | 507 | 437 | 70 | 2 | 0.46 | 6 | 2.62 |
| FQa | 60 | 73 | 67 | 6 | 3 | 0.28 | 7 | 2.23 |
| FQb | 70 | 83 | 70 | 13 | 5 | 0.32 | 12 | 4.38 |
| FQc | 19 | 14 | 13 | 1 | 2 | 0.24 | 2 | 1.22 |
| LAa | 107 | 182 | 157 | 25 | 6 | 0.36 | 15 | 5.86 |
| Lab | 114 | 531 | 365 | 166 | 5 | 0.45 | 9 | 3.32 |
| Lac | 96 | 244 | 172 | 72 | 2 | 0.35 | 10 | 3.43 |
| QZa | 90 | 201 | 137 | 64 | 3 | 0.30 | 8 | 3.50 |
| QZb | 116 | 436 | 379 | 57 | 4 | 0.41 | 8 | 3.43 |
| QZc | 124 | 279 | 260 | 19 | 9 | 0.51 | 12 | 5.58 |
| ZXa | 97 | 330 | 307 | 23 | 7 | 0.48 | 10 | 3.74 |
| ZXb | 107 | 709 | 585 | 124 | 4 | 0.51 | 8 | 2.89 |
| ZXc | 117 | 741 | 571 | 170 | 3 | 0.49 | 6 | 2.60 |
| JOa | 108 | 201 | 185 | 16 | 7 | 0.38 | 13 | 5.78 |
| JOb | 147 | 348 | 313 | 35 | 10 | 0.53 | 13 | 4.79 |
| JOc | 141 | 1039 | 643 | 396 | 4 | 0.49 | 10 | 2.99 |
| CTa | 107 | 250 | 203 | 47 | 7 | 0.47 | 9 | 3.66 |
| CTb | 108 | 220 | 159 | 61 | 10 | 0.50 | 14 | 5.27 |
| CTc | 80 | 127 | 99 | 28 | 6 | 0.29 | 10 | 3.36 |
| HYa | 112 | 263 | 221 | 42 | 7 | 0.41 | 14 | 5.14 |
| HYb | 112 | 456 | 384 | 72 | 5 | 0.40 | 10 | 3.74 |
| HYc | 79 | 122 | 77 | 45 | 3 | 0.53 | 8 | 3.57 |
| QXa | 107 | 245 | 162 | 39 | 7 | 0.39 | 12 | 4.68 |
| QXb | 95 | 235 | 397 | 39 | 6 | 0.46 | 10 | 3.67 |
| QXc | 88 | 224 | 215 | 64 | 2 | 0.33 | 8 | 2.96 |
| HKa | 86 | 153 | 125 | 28 | 6 | 0.33 | 7 | 3.17 |
| HKb | 76 | 88 | 79 | 9 | 4 | 0.19 | 12 | 4.82 |
| HKc | 103 | 344 | 291 | 53 | 5 | 0.33 | 10 | 3.57 |

**Table S3** Partial mantel test between network topology attributes and variables

| Parameter | Control for | Network topology properties | |
| --- | --- | --- | --- |
|  |  | R | *P* |
| MAT | Richness index | 0.254 | 0.004 |
| Richness index | MAT | -0.004 | 0.472 |

Network topology properties: node number; edge number; modularity; positive correlations; negative correlations; average clustering coefficient; network diameter; characteristic path length.

**Table S4** Changes of network topology attributes with latitude.

|  | Slope | R^2^ | P |
| --- | --- | --- | --- |
| Node number | -2.61 | 0.441 | <0.001 |
| Edge number | -10.40 | 0.149 | 0.011 |
| Positive correlations | -8.77 | 0.185 | 0.007 |
| Negative correlations | -2.16 | 0.065 | 0.014 |
| Modularity | -0.140 | 0.222 | 0.002 |
| Network diameter | -0.005 | 0.130 | >0.05 |
| Average clustering coefficient | -0.147 | 0.156 | 0.021 |
| Characteristic path length | -0.057 | 0.161 | 0.015 |

**Table S5** Information of 33 keystone OTUs involved in common co-occurrence relationship.

| Name | Class | Order | Family | Genus | Species |
| --- | --- | --- | --- | --- | --- |
| OTU1 | Methanomicrobia | Methanomicrobiales | Methanoregulaceae | Methanoregula | / |
| OTU2 | Methanobacteria | Methanobacteriales | Methanobacteriaceae | Methanobacterium | / |
| OTU5 | Methanomicrobia | Methanosarcinales | Methanosarcinaceae | Methanosarcina | / |
| OTU6 | Methanomicrobia | Methanosarcinales | Methanosarcinaceae | Methanosarcina | uncultured_Methanomicrobia_archaeon |
| OTU12 | Methanomicrobia | Methanosarcinales | Methanotrichaceae | Methanothrix | / |
| OTU14 | Methanomicrobia | Methanomicrobiales | Methanoregulaceae | Methanoregula | Candidatus_Methanoregula_boonei |
| OTU17 | Methanomicrobia | Methanocellales | Methanocellaceae | Methanocella | uncultured_Methanomicrobia_archaeon |
| OTU24 | Methanobacteria | Methanobacteriales | Methanobacteriaceae | Methanobacterium | / |
| OTU31 | Methanomicrobia | Methanomicrobiales | Methanoregulaceae | Methanoregula | / |
| OTU37 | Methanomicrobia | Methanosarcinales | Methanotrichaceae | Methanothrix | / |
| OTU38 | Methanobacteria | Methanobacteriales | Methanobacteriaceae | Methanobacterium | / |
| OTU47 | Methanobacteria | Methanobacteriales | Methanobacteriaceae | Methanobacterium | Methanobacterium_lacus |
| OTU64 | Methanomicrobia | Methanosarcinales | Methanotrichaceae | Methanothrix | / |
| OTU77 | Methanomicrobia | Methanosarcinales | Methanotrichaceae | Methanothrix | uncultured_archaeon_OS-18 |
| OTU89 | Methanomicrobia | Methanomicrobiales | / | / | / |
| OTU97 | Methanomicrobia | Methanosarcinales | Methanotrichaceae | Methanothrix | uncultured_archaeon_OS-18 |
| OTU165 | Methanomicrobia | Methanosarcinales | / | / | / |
| OTU174 | Methanomicrobia | Methanosarcinales | / | / | / |
| OTU256 | Methanomicrobia | Methanomicrobiales | Methanoregulaceae | Methanoregula | uncultured_Methanomicrobia_archaeon |
| OTU340 | Methanomicrobia | Methanocellales | Methanocellaceae | Methanocella | / |
| OTU367 | Methanomicrobia | Methanocellales | Methanocellaceae | Methanocella | Methanocella_paludicola_SANAE |
| OTU442 | Methanomicrobia | Methanosarcinales | / | / | / |
| OTU448 | Methanomicrobia | Methanomicrobiales | / | / | / |
| OTU476 | Methanomicrobia | Methanosarcinales | / | / | / |
| OTU518 | Methanomicrobia | Methanomicrobiales | Methanoregulaceae | Methanoregula | Methanoregula_formicica_SMSP |
| OTU519 | Methanomicrobia | Methanomicrobiales | Methanoregulaceae | Methanoregula | uncultured_Methanomicrobia_archaeon |
| OTU593 | Methanomicrobia | Methanocellales | Methanocellaceae | Methanocella | Methanocella_paludicola_SANAE |
| OTU856 | Methanomicrobia | Methanocellales | Methanocellaceae | Methanocella | / |
| OTU1100 | Methanomicrobia | Methanosarcinales | / | / | / |
| OTU1136 | Methanomicrobia | Methanosarcinales | / | / | archaeon_enrichment_culture_clone_LCB_A1C7 |
| OTU1148 | Methanomicrobia | Methanosarcinales | / | / | archaeon_enrichment_culture_clone_LCB_A1C7 |
| OTU1164 | Methanomicrobia | Methanocellales | Methanocellaceae | Methanocella | Methanocella_paludicola_SANAE |
| OTU1208 | Methanomicrobia | Methanosarcinales | Methanotrichaceae | Methanothrix | / |
